# Building a Mobile Stroke Unit Based on 5G Technology – A Study Protocol

**DOI:** 10.3389/fphys.2021.752416

**Published:** 2021-11-25

**Authors:** Gangfeng Gu, Junyao Jiang, Bo Zheng, Xiao Du, Ke Huang, Qinfang Yue, Jian Wang

**Affiliations:** Department of Neurology, Ya’an People’s Hospital, Ya’an, China

**Keywords:** stroke treatment, 5G technology, randomized controlled trial (MeSH), utility weighted mRS, study protocol

## Abstract

**Background:** In-time treatment of acute stroke is critical to saving people’s lives and improving the quality of post-stroke life. A mobile stroke unit (MSU) with fifth-generation (5G) mobile networks strengthens the interaction of patient information and healthcare resources, thereby reducing response times and improving thrombolysis results. However, clinical evidence of better outcomes compared to regular care is still lacking.

**Method and Design:** In this randomized controlled trial, 484 patients with acute stroke are allocated into the MSU and regular care groups. We establish medical records for each patient and conduct a follow-up of 90 days. The primary outcomes are functional results as defined by utility-weighted modified Rankin Scale (uw-mRS) 90 days after the incidence occurred, whereas secondary outcomes include the alarm to CT scan completed time, the alarm to treatment decision time, the alarm to thrombolytic time, quality of life, and symptomatic intracranial hemorrhage combined with NIHSS score as well as cost-effectiveness.

**Discussion:** This study establishes an innovative MSU (based on 5G) to manage acute stroke, comparing its clinical and economic outcomes to regular care and informing decision-makers of the effectiveness of the stroke emergency system.

**Clinical Trial Registration:** [http://www.chictr.org.cn/showproj.aspx?proj=63874], identifier [ChiCTR2000039695].

## Introduction

Patients with acute stroke require medical aid at the first moment to quickly work out suitable treatment options, thus minimizing adverse results after stroke (Peisker et al., 2017). Clinical guidelines propose that prehospital treatment, including ambulance arrangement and tissue plasminogen activator (t-PA) treatment, is essential to saving patients with acute stroke and improving their quality of life (Powers et al., 2018, 2019). To this end, an emergency medical system (EMS) with a quick response to stroke calling and well-trained emergency personnel is needed. Emergency experts have been searching for innovative approaches that transcend the conventional pathway to provide patients with better medical services. A mobile stroke unit (MSU) is a special ambulance that incorporates neurological examination, CT diagnosis, and intravenous thrombolytic therapy by fitting a mobile CT and relevant point-of-care laboratory testing (Bowry and Grotta, 2017; Tsivgoulis et al., 2018).

As a timely administration of thrombolytic therapy, MSU has apparent benefits for acute stroke patients (Nyberg et al., 2018; Yamal et al., 2018). In China, the MSU equipped with a mobile CT was introduced in 2017, effectively reducing the “door to needle” from over 60 min to around 48 min. As a result, hospitals have established their MSUs across the country, in efforts to elevate the local medical services and social welfare (Zhou et al., 2021). Moreover, with the recent universalization of fifth-generation (5G) technology, the MSU has gradually transformed into a new model by equipping with the latest mobile networks and state-of-the-art monitoring devices, balancing essential functions of EMS and other advanced gadgets. Therefore, the highly informative and intelligent MSU enables rapid treatment of acute stroke, reducing stroke patients’ disability and death rates and the economic burden on their family as well as the entire society. Furthermore, the highly integrated wearable devices could acquire the core medical data (including vital signs, cranial pressure monitoring, blood glucose, etc.) in real time, which is simultaneously uploaded to a stroke-specific cloud platform for remote consultation and better surveillance.

## Methods and Analysis

### Study Design

**FIGURE 1:**
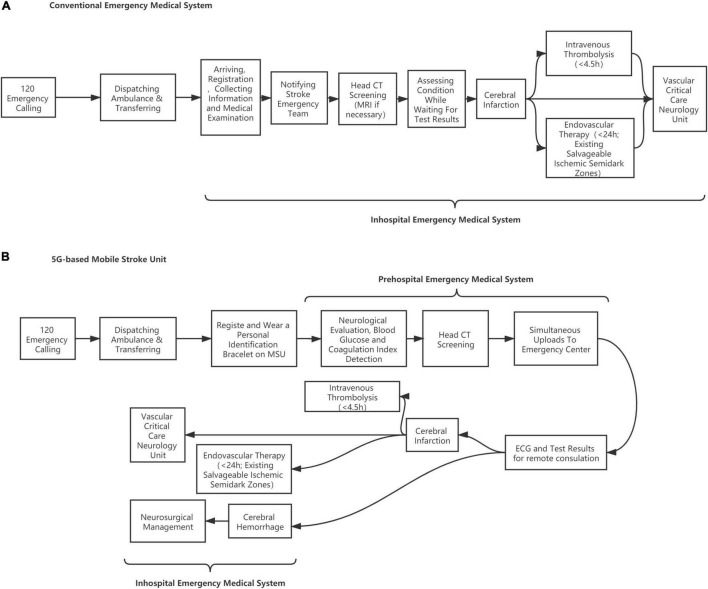
Flow diagram of patients screening. **(A)** Conventional emergency medical system. **(B)** 5G-based mobile stroke unit.

We conduct a pragmatic, prospective trial to examine clinical and economic outcomes in patients eligible for thrombolytic administration who receive care from MSU, as compared with regular care in a blinded fashion (see Figure 1), determining. Through this, we aim to determine whether the deployment of MSU leads to better functional benefits for patients with acute stroke. Investigators randomly allocate one patient to either the MSU or the regular care group. To balance the two groups on potential confounding factors (such as weather) that may change in a year, we choose a block size of 4 weeks to limit the length of time to use the same program.

The protocol that is registered with No. ChiCTR2000039695 on 5 November 2020 (http://www.chictr.org.cn/showproj.aspx?proj=63874) has been approved by the ethical review committee of the Ya’an people’s Hospital (NPSY202007002).

### Participants

A total of 484 participants are recruited from the Ya’an people’s Hospital, 242 of whom were randomized to the intervention group and the rest randomized to the comparison group.

Each participant must meet all of the inclusion criteria to be enrolled in this study, which are given as follows:

1. Patients need to be aged over 18 years, with a final diagnosis of ischemic stroke.
2. Patients’ confirmed onset-to-alarm time is less than 4.5 h.
3. Patient is recognized as a t-PA candidate, with mRS scores less than 3.
4. Patients volunteer to participate and sign an informed consent.

The exclusion criteria include uncertain symptom onset, no focal stroke-like symptoms, severe malignant, or pregnancy.

### Intervention and Comparison

**FIGURE 2:**
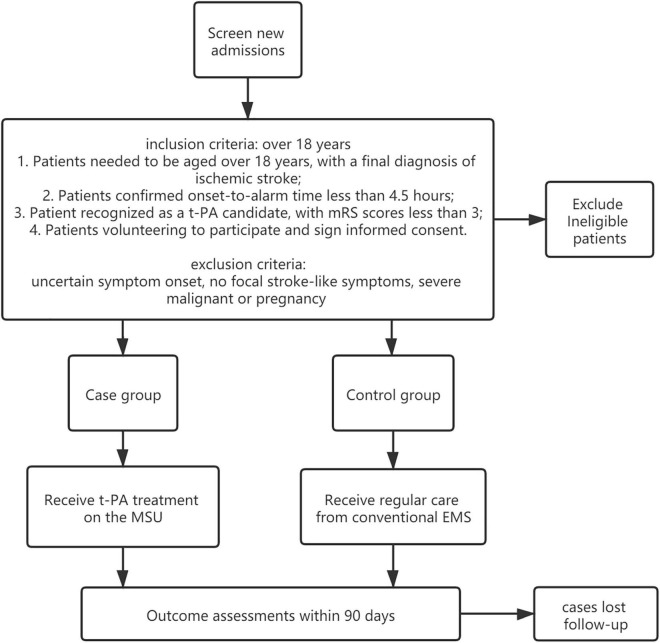
Regular care life chain versus 5G-based SSES life chain.

The case group is treated in the MSU, which is designed to establish a treatment unit integrating neurological symptom examination and CT diagnosis, achieving intravenous thrombolytic therapy and remote consultation at the first moment. During transportation, the physician can carry out neurological evaluation (NIHSS scores), blood glucose, coagulation index detection, and head CT scanning on the ambulance where data are transmitted to the stroke center in real time through 5G networks for technical support and treatment guidance from experts. Once the patient is recognized as suitable for thrombolysis, t-PA treatment will be given within the “golden time” of stroke management. The MSU team includes a paramedic, a stroke doctor, and a neuroradiologist, integrating the conventional EMS in a mixed urban–rural environment. The patient is evaluated for vital signs and neurological impairment followed by head CT screening and blood biochemical examination. The patients who meet the requirements of intravenous thrombolysis will be given t-PA treatment on the MSU vehicle after the informed consent is signed. Those who are not suitable for intravenous thrombolysis will be transported to the stroke center for further treatment. If necessary, remote consultation will be carried out through a customized telemedicine system based on 5G technologies. The MSU adopts a 7-day 24-h working system. In contrast, the control group is treated through conventional EMS in which the city emergency medical rescue center dispatches an ordinary ambulance. Head CT screening, blood biochemical examination, and the followed t-PA treatment (if suitable) will be performed after patients are delivered to the hospital’s emergency center (Nor et al., 2005) (see Figure 2).

### Primary and Secondary Outcomes

The primary endpoint is the utility-weighted modified Rankin Scale (uw-mRS) at 90 days assessed with a telephone interview. Secondary endpoints include time-dependent phenomena such as the alarm to CT scan completed time, the alarm to treatment decision time, the alarm to thrombolytic time, quality of life assessed with EQ-5D at follow-ups over 5 years, symptomatic intracranial hemorrhage combined with NIHSS score, as well as cost-effectiveness.

### Sample Size Estimation

Previous studies suggested that the difference in mean 90d uw-mRS between groups ranged from 0.024 to 0.25. However, according to our pilot study data, we anticipate that the difference in 90d mean uw-mRS is 0.09 (0.59 vs. 0.50) with a standard deviation of 0.5 in both groups. Considering a type-I error of 0.05, a study power of 0.8, and a two-sided test, we included a total sample size of 484 participants after considering a 10% dropout rate, each group with 242 participants.

### Statistical Analysis

We calculate the sample size by comparing two independent samples. A *t*-test compares the mean uw-mRS at 90 days and corresponding two-sided 95% confidence intervals if the assumption of normality is not valid. Although the mRS is a primary endpoint, the difference among the uw-mRS categories is significant, and the *t*-test assumption and central limit theorem are probably met. The calculation of uw-mRS will be adjusted for baseline uw-mRS and baseline covariates associated with mRS in a linear regression model. Stratified analyses of the primary outcome and pooled measures are conducted by the Cochran–Mantel–Haenszel analyses and multiple ordinal regressions. We use a proportional odds model to process dichotomized outcomes of uw-mRS 0-1 vs. 2-6 with binary logistic regression. Participants in the intervention group who give up during the program will be excluded from the analysis. In addition, subjects in both treatment groups will be excluded from the protocol analysis if any violations of the following protocols after randomization and during intervention and follow-up. All analyses are performed by SPSS 27.0.

Besides, we explore the missing data and compare the distribution of critical variables. Suppose there is a reasonable amount of missing data and the data summary indicates that the data are missing at random. In that case, all analyses will be performed after multiple imputations of missing data using baseline variables as auxiliary variables.

## Discussion

This study explores whether stroke patients clinically benefit from MSU deployment at 90 days. Given that many countries have encountered problems resulting from the increasing elderly population and related burden of cerebrovascular diseases, this finding could project some hope on emergency care based on the latest mobile networks and state-of-the-art devices to relieve stroke-related problems. As an expensive and complicated intervention, prehospital stroke management presents different models globally, making its clinical outcomes and cost-effectiveness difficult to evaluate, thus limiting its widespread utilization. Although some famous research works about the MSU gain better results about the clinical application (Kunz et al., 2016; Ebinger et al., 2021), the evidence is insufficient due to the limited sample size, unstandardized disposition of devices, and economic conditions of varied regions in the world. Therefore, a prospective controlled trial based on China’s experience can provide healthcare decision-makers with clinical and administrative data in developing countries. However, selection bias by distributing patients according to MSU availability is hardly avoided, potentially causing the baseline information to be divided in the end. Second, the accurate data of uw-mRS are hardly accessed through telephone interviews. Therefore, we try to eliminate the offset by conducting multi-dimensional follow-ups through the Wechat survey applet. Third, a potential challenge is that it is difficult to identify patients with suspected stroke to suitable distribution in the dispatch center after an emergency call was made, thus making the prehospital stroke management unaccessible. Limited availability weakens a clear comparison group with all critical factors of a prospective study where MSU is compared to regular care. Overall, potential bias will be minimized by operationalized standards and adjustment for baseline parameters.

## Patient and Public Involvement

Acute stroke patients are recruited for this research. The dissemination plan for the overview includes promotion via social media, presentation at conferences, and dissemination to patient advocacy groups.

## Ethics Statement

The studies involving human participants were reviewed and approved by Ya’an People’s Hospital (Approval No. 2020-24). The patients/participants provided their written informed consent to participate in this study.

## Author Contributions

JW provided the idea of this research and wrote the draft protocol. GG and BZ completed the details of the protocol. KH, QY, and JJ participated in the study design and literature review. XD was responsible for statistical analysis. All authors contributed to the article and approved the submitted version.

## Conflict of Interest

The authors declare that the research was conducted in the absence of any commercial or financial relationships that could be construed as a potential conflict of interest.

## Publisher’s Note

All claims expressed in this article are solely those of the authors and do not necessarily represent those of their affiliated organizations, or those of the publisher, the editors and the reviewers. Any product that may be evaluated in this article, or claim that may be made by its manufacturer, is not guaranteed or endorsed by the publisher.
